# Illustrating psychometric tests, scales, and constructs: An R package for Item Pool Visualization

**DOI:** 10.3758/s13428-022-02052-7

**Published:** 2023-02-07

**Authors:** Nils Petras, Michael Dantlgraber, Ulf-Dietrich Reips

**Affiliations:** 1 School of Social Sciences, University of Mannheim, L 13, 15, 68161, Mannheim, Germany; 2 Department of Psychology, University of Konstanz, Konstanz, Germany; 3 Kalaidos University of Applied Sciences, Zürich, Switzerland

**Keywords:** Item Pool Visualization, Construct validity, Nomological network, Factor structure, Psychological assessment

## Abstract

Researchers assessing psychological constructs have to understand and choose between several competing measures. Item Pool Visualization (IPV, Dantlgraber et al., 2019) was developed to offer a systematic and detailed portrayal of the actual content and internal balance of competing measures. To enable the use of IPV, we developed and present here the IPV R package. Its aim is to allow researchers to add IPV to their repertoire with minimal effort. Creating IPV charts from raw data requires two simple function calls, because the package streamlines model specification, model estimation, and chart creation. It improves IPV conceptually by introducing the aggregate center distance and the item overview chart. It provides many customization options and generates high-quality, vector-based PDF output. The workflow of the package is explained using a reproducible open data example from a personality assessment.

Researchers assessing psychological constructs routinely choose between several competing measures. Available measures differ in both their psychometric quality and—importantly—their content. This can be difficult, because the abstract labels of the measures rarely provide sufficient information about their precise content. Firstly, different content is frequently labeled very similarly (jingle-fallacy) and very similar content is frequently labeled differently (jangle-fallacy) (Block, 1995; Leising et al., 2021). For example, “agreeableness” in the Big Five model is conceptually and empirically distinct from “agreeableness” in the HEXACO model (Ashton & Lee, 2019; Ashton et al., 2014). In another example, the “Sports Mental Toughness Questionnaire” turned out to be empirically more similar to two “Self-Esteem” measures than these were similar to each other (Dantlgraber et al., 2019). Secondly, the integrated content of multidimensional measures is usually unbalanced (e.g., Hulleman et al., 2010). In many cases, some parts of the measure define the meaning of its overall score much more than other parts. The defining parts are represented by more items or more strongly correlated items than other parts of the measure. We argue that the true content behind vague terminology and the internal imbalance of measures can be difficult to grasp by standard procedures. Nevertheless, the choice of measurement instruments in psychology should take these aspects of their substantive internal structure into account. Ultimately, the goal is to select the most appropriate measure for the research question at hand—and to provide strong arguments to justify the choice (Flake & Fried, 2020).

## Item Pool Visualization

To offer a systematic and detailed portrayal of the actual content and internal balance of competing measures, *Item Pool Visualization* (IPV) was developed (Dantlgraber et al., 2019). In IPV, an *item pool* is a large set of correlated items (manifest indicator variables) that are assessed together. To map the content within the item pool, it can be split repeatedly into smaller sub-pools. Each item can then be represented by a general term describing the whole item pool (e.g., “intelligence”) or by increasingly specific terms describing sub-pools (e.g., “memory capacity”, “verbal memory capacity”). Because the total item pool may include any combination of correlated measures, IPV is well suited to analyze and compare multiple competing measures together, shedding light on the jingle-jangle jungle.

**Fig. 1:**
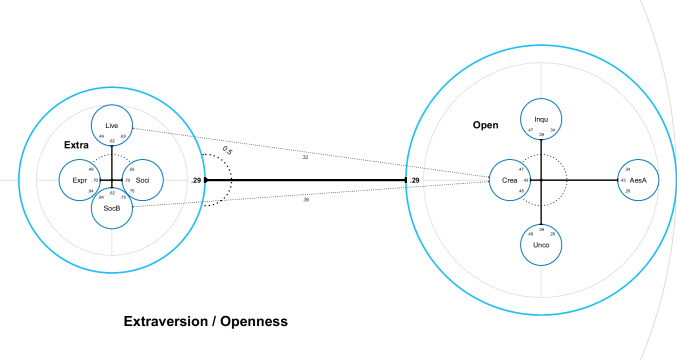
Nested chart of Extraversion (Extra) and Openness (Open), cropped at the left, top, and bottom for convenient display; AesA = Aesthetic Appreciation, Inqu = Inquisitiveness, Crea = Creativity, Unco = Unconventionality, Soci = Sociability, Live = Liveliness, Expr = Expressiveness, SocB = Social Boldness

Item Pool Visualizations are radially structured representations of measures (Fig. 1 shows the example discussed below). Using the distance to the center, IPV illustrates which item sub-pools (or individual items) constitute the core of the item pool and which farther ones are less representative. Items that are represented centrally are equally well described by general and specific terms, while the description of items far from the center profits immensely from the distinction of sub-pools. The most specific sub-pools should represent homogeneous and reliable scales. This is usually established in the original publication of a scale. IPV provides an immediate impression of the internal balance and diversity of the item pool that is hard to deduce from a standard path diagram of a structural equation model (SEM, see e.g., Kline, 2015). When it is used to display one large item pool consisting of multiple competing measures of a psychological construct, IPV allows a direct and detailed comparison of the measures’ content and structure.

### How to read an IPV chart

1. Circles represent item pools. Circles within other circles represent subordinate item pools (e.g., facets of a personality trait such as the four facets of Extraversion on the left of Fig. 1).
2. Numbers represent latent correlations between item pools at the same level of specificity and are displayed in order (pairwise opposite each other). Dotted arrows are drawn if a correlation exceeds that of the superordinate pools, marking an additional commonality (e.g., the correlation between Creativity and Social Boldness in Fig. 1 exceeds that of Extraversion and Openness: .36 > .29).
3. The distances from the center of a circle to the edges of smaller subordinate circles (=center distances, black bars) show the internal balance of the item pool. The defining parts of the measure are more centered: The distance from the center increases with the relative increase in factor loadings when using more specific terminology (=factors, smaller circles). The scale is given by the central dotted circle.
4. If there are several subdivisions, there is a route of center distances from the most general model (surrounding largest circle, gray) to the most specific model (smallest circles). The first section is the distance between the overall center to the edge of a circle (light blue “Extra” or “Open” in Fig. 1). The route continues from the center of this smaller circle, which then “zoomed in” becomes the new reference item pool for further subdivision.

A more detailed example is discussed below (see also Dantlgraber et al., 2019, p. 7).

### Advantages of IPV

IPV adds to standard SEM analysis by comparing the factor loading estimates of multiple SEMs, beyond the analysis of a single model. It combines the strengths of other common factor models and avoids some of their limitations:

- **Correlated-factor model.** IPV is based on a comparison of a single-factor model and one or more models with correlated factors. IPV shows the bivariate relationships between item pools as correlations on several levels of specificity. Compared to a singular correlated-factor model, IPV adds a hierarchical structure matching the common use of both total and facet-specific subscale scores.
- **Higher-order factor model.** Higher-order factor models (e.g., Kline, 2015; see also Yung et al., 1999) also introduce a hierarchical structure. They include higher-order, more general factors, which fully explain the correlational structure of the lower-order, more specific factors. Other than higher-order models, IPV offers a direct estimation of factor correlations at the same level (i.e., construct and facet relationships). In the higher-order model, these relationships cannot be seen directly, but only via an analysis of several higher-order factor loadings, which are estimated under the strong (and usually violated) assumption that higher-order factors fully explain the common variance of their respective lower-order factors. Unlike higher-order models, IPV does not need the assumption that the relationship between the more general higher-order factors and the items is mediated by interjacent factors. Therefore, IPV can account for qualitatively different item loading patterns on different levels of specificity.
- **Bi-factor model.** Similar to the bi-factor model (Holzinger & Swineford, 1937), in IPV, both general factors and specific factors for each item sub-pool (e.g., facet of a personality trait) are estimated. Unlike the bi-factor model, IPV does not restrict the estimation of factors by an orthogonality constraint. In IPV, the bivariate correlations between sub-pools are estimated directly. The bi-factor model accounts for relationships between different item sub-pools only by item-specific loadings on a common general factor, meaning that it does not model any commonality between sub-pools beyond their affiliation to the common general factor. However, analyzing the direct bivariate relationships between facets across measures is important, because it enables a proper understanding of their (partial) similarities and differences.

For these reasons, if one is interested in the question of how well a set of general and specific terms explain the sum scores of given (sub-)pools of items, we argue that IPV is preferable. In its visualization, compared to standard path diagrams of SEMs, IPV is optimized to reduce clutter due to large numbers of correlation arrows. Rather than printing many numbers in figures or tables, it represents relative sizes of factor loadings by intuitively readable positioning. It is a specialized display to compare the internal structure and balance of measures. There is no equivalent path diagram.

## The IPV R package

To enable the use of IPV, we developed and present here the IPV R package (Petras & Dantlgraber, 2022). The package eases the multistep process of estimating multiple models and combining their estimates statistically. It automates model specification based on the lavaan package (Rosseel, 2012), the subsequent retrieval of key statistics, the computation of statistical indices, and the creation of charts. It provides convenient code to display IPV charts accurately despite their complex radial design. Using the underlying ggplot2 package (Wickham, 2016) directly would be orders of magnitude more difficult and laborious. The necessary code to create high-quality IPV charts from raw data includes only two simple function calls. The package adds to the original conception of IPV (Dantlgraber et al., 2019) by improving the way center distances are aggregated across items and providing a bar chart to examine absolute (in addition to relative) increases in factor loadings. IPV enables users to quickly and thoroughly customize the appearance of the charts. The next two sections contain a more detailed description of the IPV concept, as well as a hands-on introduction to the workflow and features of the IPV R package using an example from a personality assessment.

### Statistical models

**Fig. 2:**
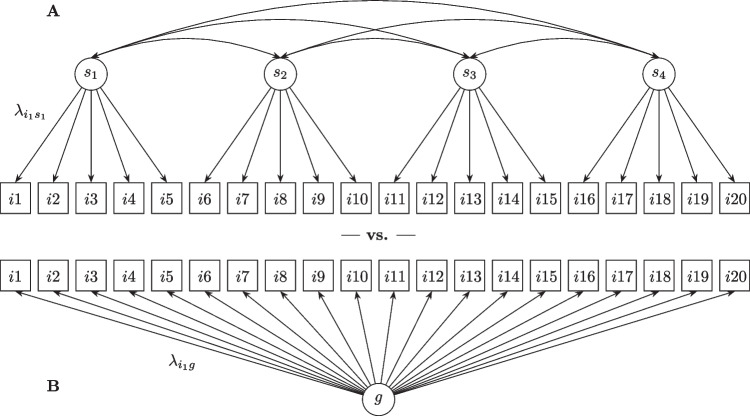
Example of IPV models: A general model (**B**) and a model using more specific factors and terms (**A**)

To analyze the internal structure of item pools, IPV compares several nested factor models of the same data. Figure 2 shows an example path diagram of two confirmatory factor models.

Model B represents all items by one factor, whereas Model A splits the total item pool into four item sub-pools, represented by four correlated factors. Note that these sub-pools could be split again, resulting in a third model with even more correlated factors. IPV is usually based on the comparison of two or three of these models: The *center distance* statistic (*cd*) is used to quantify the degree to which a specific term (e.g., Model A) describes an item better than a general term (e.g., Model B). Equation 1 shows how center distances are calculated from the factor loading estimates of the models.

1 $$c{d}_i=\left\{\begin{array}{ll}{\lambda}_{is}^2/{\lambda}_{ig}^2-1&\ \textrm{if}\ {\lambda}_{is}/{\lambda}_{ig}>1\\ {}0&\ \textrm{if}\ {\lambda}_{is}/{\lambda}_{ig}\le 1\end{array}\right.$$

The center distance indicates the relative amount of additional explained item variance by the more specific factor (e.g., *s*_1_) compared to the more general factor (e.g., *g*). For example, an item with the factor loadings *λ*_*g*_ = 0.4 and ${\lambda}_{s_1}=0.8$ has a center distance of 0.8^2^/0.4^2^ − 1 = 3. Negative center distances are set to 0 for display.

- **Aggregate center distances.** Center distances of multiple items in an item pool can be summarized by computing the *mean center distance* (Dantlgraber et al., 2019). Here we propose the *aggregate center distance* of an item pool as a more refined statistic (Eq. 2). We argue that it yields a more straightforward and more intuitive interpretation. The difference is largest when some center distances can be identified as outliers because of very low factor loadings (in comparison to other items from the item pool).

2 $$c{d}_k=\frac{\sum_{i=1}^m{\lambda}_{is}^2}{\sum_{i=1}^m{\lambda}_{ig}^2}-1$$

The aggregate center distance *cd*_*k*_ of the specific item pool *s*_*k*_ is the relative increase in explained item variance across all its *m* items. Its interpretation is analogous to the interpretation of the center distance of a single item^1^. Consider the following example: Be *x* and *y* two items for which *λ*_*xg*_ = .1, *λ*_*xs*_ = .2, *λ*_*yg*_ = .6, and *λ*_*ys*_ = .8. It follows from Eq. 1 that *cd*_*x*_ = 3 and *cd*_*y*_ = 0.78. The increase of the explained item variance of *x* from 0.1^2^ = 1% to 0.2^2^ = 4% produces a high mean center distance of (3 + 0.78)/2 = 1.89. This may lead to the erroneous conclusion that *s* explains 189% more overall item variance than *g*. It seems counterintuitive to obtain such a large value. The high relative but small absolute increase on *x* barely contributes to the overall explained item variance of the item pool. The aggregate center distance, on the other hand, directly represents the relative increase in explained overall item variance: *cd*_*k*_ = (0.04 + 0.64)/(0.01 + 0.36) − 1 = 0.84 = 84%. Furthermore, the aggregate center distance accurately determines the net gain in explained item variance, even if some items show a loss (*λ*_*is*_ < *λ*_*ig*_). Both ways of calculating center distances are available in the package.

### Use of the IPV package

**Fig. 3:**
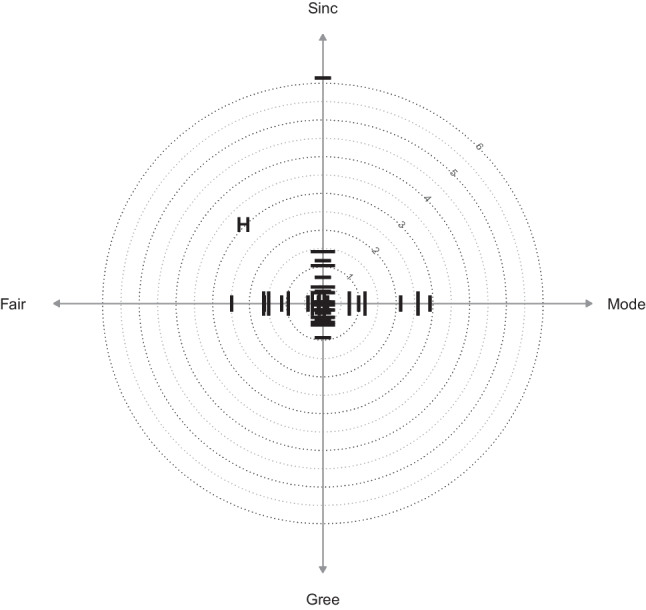
Item chart of honesty/humility (H) items; Sinc = Sincerity, Fair = Fairness, Gree = Greed Avoidance, Mode = Modesty

**Fig. 4:**
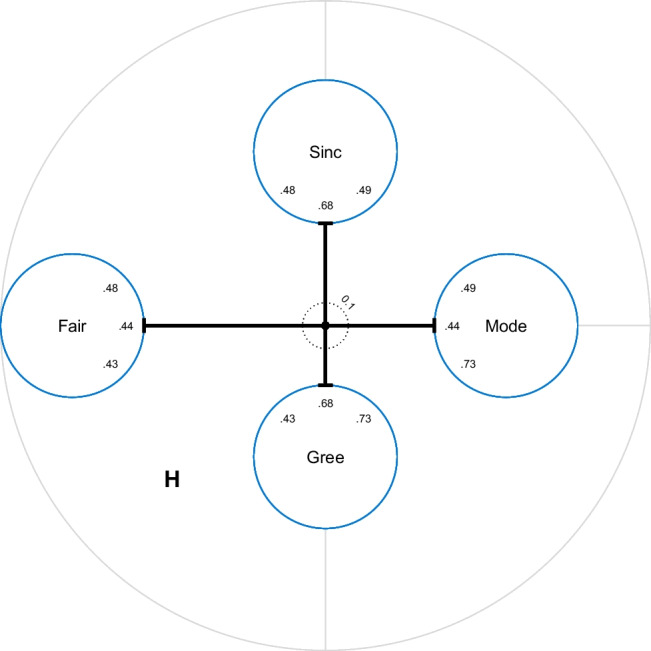
Facet chart of honesty/humility (H) items; Sinc = Sincerity, Fair = Fairness, Gree = Greed Avoidance, Mode = Modesty

Based on these center distance statistics, the R package IPV provides three different types of charts: item charts, facet charts, and nested charts. *Item charts* show the center distances of individual items, grouped by their respective item sub-pool (see Fig. 3). They are most useful to inspect content variation within item sub-pools and identify outlier items. *Facet charts* show the aggregate (or mean) center distances of item sub-pools (see Fig. 4). They include correlations between the factors representing item sub-pools. Facet charts are most useful to inspect the internal structure of measures. For complex cases with two subdivisions of the item pool, *nested charts* combine multiple facet charts (see Fig. 1). They are most useful to compare multiple measures and their internal structure in one large analysis. The following section on the R package IPV provides a more detailed explanation of both the charts and the example.

The package IPV enables users to estimate and plot IPV models. It uses raw data as input to automatically specify and estimate factor models. Center distances and other relevant information is automatically generated for direct use by the plot functions of the package. On the one hand, the plot functions provide users with options for a thorough customization of the appearance of the charts. On the other hand, they can be called using only the input data for instant results. In this way, an IPV chart is just two simple function calls away from the raw data (see below). The package provides several functions to create high-quality figures for publication, such as colored, vector-based PDF graphics files of sections of the charts.

Model estimation is based on lavaan (Rosseel, 2012) and plots are created from elements provided by ggplot2 (Wickham, 2016) and ggforce (Pedersen, 2020). Users can opt to provide their own model estimates instead of raw data, or use the center distances without plotting them. The package can be accessed via the CRAN (Version 1.0.0, Petras & Dantlgraber, 2022) with the latest development version available on GitHub (https://github.com/NilsPetras/IPV).

After installing R (https://www.r-project.org/), the package IPV can be installed and loaded as follows:

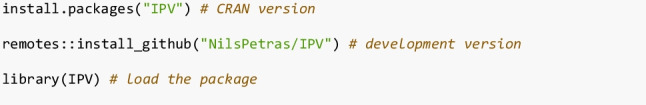



A minimal code example that creates an IPV chart from raw data could look like this:

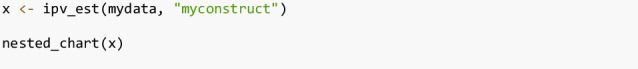



It follows a detailed introduction to the package workflow and features using empirical data.

### HEXACO example

For demonstration purposes, open data from the Open-Source Psychometrics Project (https://openpsychometrics.org/_rawdata/ retrieved May 6th, 2020, dated June 21st, 2014) is used in the following. The 240 items of the International Personality Item Pool (IPIP) HEXACO Equivalent Scales (Ashton et al., 2007) were administered by the Open-Source Psychometrics Project to an ad hoc online sample of 22,786 participants. After removing cases with any value below “agree” on the seriousness check items (Aust et al., 2013; Reips, 2009), data of 20,365 participants remained. In the treatment of the 191 cases with missing data, we relied on the default of IPV and lavaan, which is listwise deletion. Other options^2^ can be directly passed to lavaan. This data example is included in the IPV R package, so the code can be run as presented after installing and loading the package to reproduce the example.

The IPIP HEXACO Equivalent Scales consist of 40 items for each HEXACO personality trait: Honesty/Humility (H), Emotionality (E), Extraversion (X), Agreeableness (A), Conscientiousness (C), and Openness (O). Each trait has four subdomains, so that the item pool of each trait consists of four sub-pools of ten items each. The HEXACO traits are commonly thought to reflect separate constructs in a “six-dimensional structure of personality characteristics” (Ashton et al., 2004, p. 365). Nevertheless, there are two trait correlations of medium size (*r*_*HA*_ = 0.45, 95% CI [0.44, 0.46], *r*_*OX*_ = 0.30, 95% CI [0.29, 0.31]).

This raises the question of what the internal structure of these traits looks like, and exactly how their item pools relate to each other. How much better than a single factor model across all items does a model with two correlated factors for Honesty/Humility (H) and Agreeableness (A) describe the items? How much better does a model that uses the specific facets of each personality trait describe the items? Do the more specific models describe all items better, or just some?

In the following, we compare three different models of the same example data on H and A. Model 1 represents all items of H and A by a single factor. Model 2 uses two correlated factors: one for H and one for A. Model 3 uses one factor for each facet of H and A, resulting in eight correlated factors. Based on these three models, IPV charts can be created that display the internal structure of the HEXACO H and A items. IPV charts help researchers understand the structure and unique mixture of content of the IPIP HEXACO Equivalent Scales.

### Workflow

- **Model specification and estimation.** IPV automatically specifies and estimates the relevant models from the raw data, using the function ipv_est (est = estimation). The first argument (dat) is the dataset that contains the observed variables. The code in the square brackets reduces the full dataset HEXACO to the subset of items measuring Honesty/Humility (H) and Agreeableness (A) via a regular expression (grep). The second argument (name) is the name given to the total item pool in the dataset.

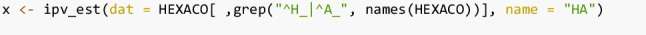

Instead of having the user specify all models, the ipv_est function conveniently infers them from the variable names. When the wide format is chosen, the only information needed is raw data with variable names in the format “test_facet_item”^3^. The data may not include any additional variables, because all variables will be used for model specification. Consider this small excerpt of the example dataset:

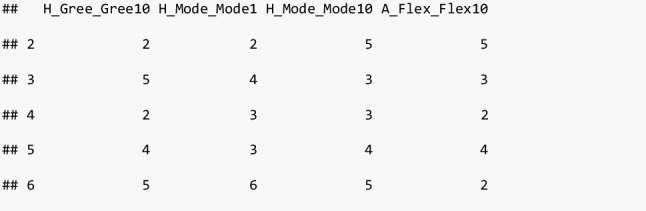



Model 1 specifies one factor across all given items. Here, only the items for H and A were provided to ipv_est. Therefore, the factor across all items is called name = "HA" in the ipv_est function call. Model 2 specifies one factor per unique label in the first part of the item names (“test”). Here, the two factors “H” and “A” are specified. Model 3 specifies one factor per unique label in the second part of the item names (“facet”). Here, eight factors are specified representing eight facets of H and A. These include Greed Avoidance (“Gree”), Modesty (“Mode”), and Flexibility (“Flex”). The third part of the item names is the unique label of the individual items. For example, the ninth item of the Modesty subscale of H is called “Mode9”. Finally, the item name H_Mode_Mode9 represents the unique and clearly identifiable item “Mode9” that is assigned to the facet “Mode” and the superordinate factor “H”. For tests without facets, or cases with just two models, variable names in the simplified format “test_item” are needed.

ipv_est returns an object of class "IPV". This output list (here: x) includes up to four elements. The first element ($est) provides the estimated center distances and correlation matrices relevant for chart creation. In the example, estimates for the comparison of Model 1 and Model 2 ($est$global) as well as for the comparison of the Model 2 to Model 3 ($est$tests) are computed.

Consider as an example some of the global center distances ($est$global$cds). These are the center distances for the first six items of the Sincerity facet of H. They compare Model 1 (all items are described by the “HA” factor) with Model 2 (one factor for each personality trait “H” and “A”):

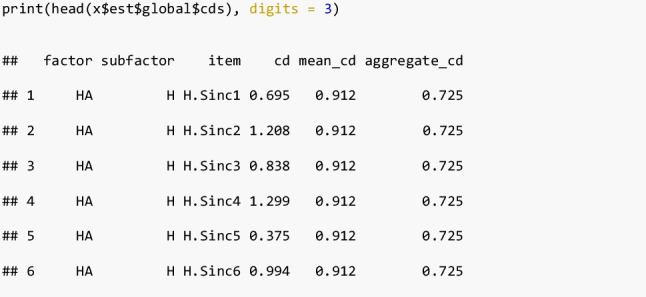



For example, the factor “H” of the more specific Model 2 explains about 70% more variance of the first Sincerity item “Sinc1” compared to the “HA” factor from the more general Model 1. Across all items of H, the increase in explained item variance using “H” instead of “HA” is about 72% (see last column).

The center distances from the comparison of Model 2 to Model 3 are stored separately for each test ($est$tests). Here, Model 2 represents the Sincerity items by a broad factor (“H”), and Model 3 represents them by a specific Sincerity factor (“Sinc”):

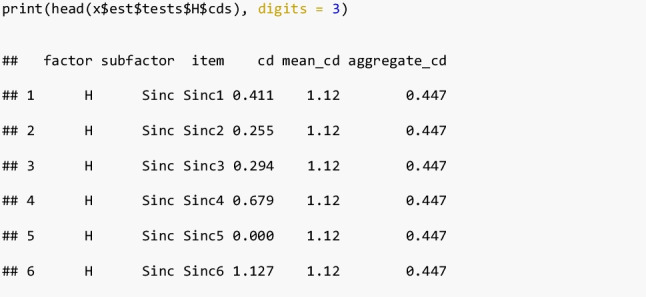



For example, the factor “Sinc” of the more specific Model 3 explains about 41% more variance of the first Sincerity item “Sinc1”, compared to the “H” factor from the more general Model 2. On average, the increase in explained item variance using “Sinc” instead of “H” is about 112% for the Sincerity items. Note the disparity between the mean center distance and the aggregate center distance due to an outlier item “Sinc10” with very low factor loadings in both models.

Finally, the first element contains the estimated latent correlations (...$cors). Displayed below are the correlations between the four facets of H ($tests$H) in Model 3:

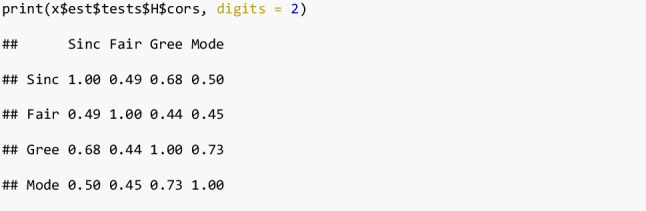



The second element ($est_raw) of the output list contains all the factor loadings in a structure similar to $est. The third element ($lav) contains the full model estimation output of all models provided by lavaan (Rosseel, 2012). The lavaan package can be used to inspect and report this output. The fourth element ($xarrow) contains cases in which the correlation of facets across tests exceeds the one between the respective tests. These are meant to be highlighted as correlation arrows in IPV nested charts (see below).

- **Chart creation.** To create an IPV chart, the output of ipv_est is simply passed to one of the three chart functions (item_chart, facet_chart, or nested_chart).

Item charts can be created for each subdivision of the item pool. They either show all items (grouped by sub-pools), or all items of a sub-pool (grouped by a further subdivision into sub-pools).

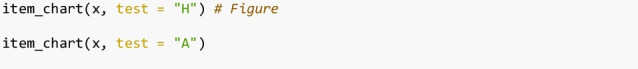



Figure 3 shows an item chart of the Honesty/Humility (H) items grouped by facets. The items of the Greed Avoidance facet (Gree) have relatively small center distances; their variance is captured only somewhat better by the factor “Gree” than by the factor “H”. The items of the other facets show much more variation in the center distances, indicating that some of their items are much better represented by their facet-specific factors than by the factor “H”. There is one particularly strong outlier on the Sincerity facet (see upper part of Fig. 3).

Facet charts can also be created for each subdivision of the item pool. They summarize the center distances of item charts by displaying aggregate center distances (default) or mean center distances. Facet charts include the latent correlations of the more specific model (by default).

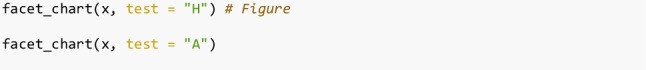



Figure 4 shows a facet chart of the H items grouped by facets. The distance from the center to the edge of the circles is the aggregate center distance of the items of the facet. Note that the Sincerity facet does not have a particularly high aggregate center distance, because the outlier item (see Fig. 3) has small factor loadings in both models (*λ*_*Sinc*10, *H*_= −0.06, *λ*_*Sinc*10, *Sinc*_= 0.16) and therefore does not fit in this questionnaire. Using mean center distances (by specifying cd_method = "mean" in the function call of facet_chart) would result in a very different picture. The latent correlations between the factors representing the four facets of H are indicated in order within the circles. Whereas the center distances describe the similarity of the four facets to the overall factor “H”, the correlations describe the similarity of the facets to each other.

Nested charts can be created from two nested subdivisions of the total item pool. They display a facet chart of the overall item pool and plug in facet charts of its sub-pools. Each circle of the overall facet chart contains another facet chart.

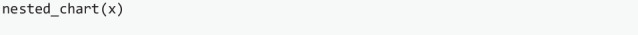



**Fig. 5:**
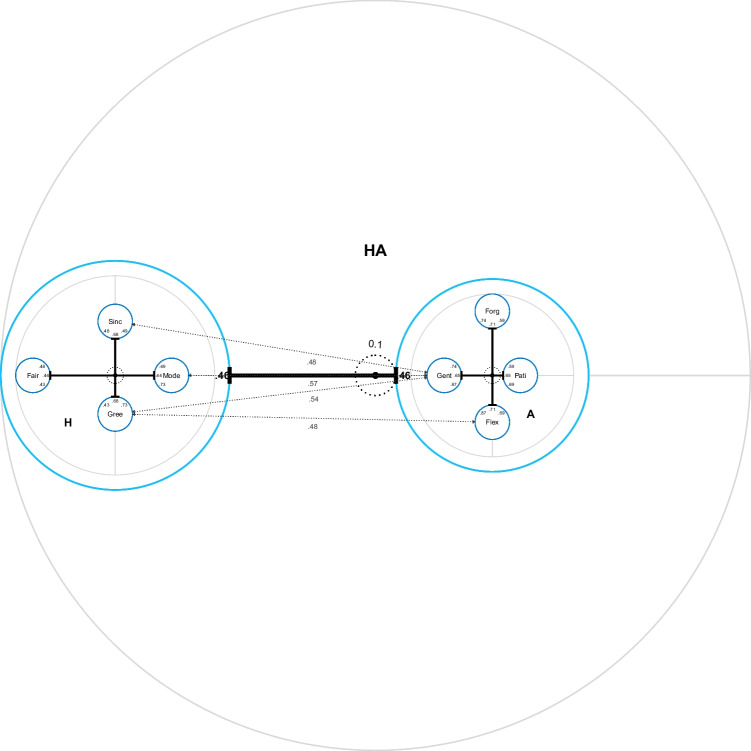
Default nested chart of the honesty/humility (H) and agreeableness (A) items; default appearance with no customization done; Pati = Patience, Forg = Forgiveness, Gent = Gentleness, Flex = Flexibility, Mode = Modesty, Sinc = Sincerity, Fair = Fairness, Gree = Greed Avoidance

Figure 5 shows a nested chart of the H and A items. It shows an overall facet chart with H and A as facets of a hypothetical overarching construct HA. The already familiar facet chart of the H items (Fig. 4) is plugged into the circle of the global H facet. Despite the potential for customization to optimize readability (see below), two things are clearly visible: First, the factor “A” is much more central to the hypothetical HA construct than the factor “H”. The meaning of the hypothetical construct would therefore be very similar to the meaning of the A items. The addition of the H items barely changes the meaning of the overall factor. Second, the facets of A are somewhat more central than those of H, which results in a smaller circle of “A” compared to “H”. This means that the facets of A are more strongly correlated with each other. Therefore, they are more similar to their overall trait A than the facets of H are to H. The higher diversity of the H measure explains why H is less potent to form a strong core of a hypothetical overarching HA construct. The additional dotted arrows across the chart indicate correlations between facets of “H” and “A”. We recommend indicating only those correlations that exceed the correlation between their superordinate item pools (here: *r*_*HA*_ = 0.46). In this way, every arrow indicates an additional similarity between facets beyond the similarity of the superordinate item pools. These arrows indicate that the facet Gentleness (Gent) is the facet of A that is most similar to the facets of H. The ipv_est function automatically provides the necessary information to add these arrows ($xarrow).

### Customization

Although default values adapt intelligently to the data, they cannot guarantee the most readable or most aesthetic result. Therefore, IPV enables users to thoroughly customize charts based on requirements and preferences. For example, it is possible to resize, rotate, and relabel chart elements. For final results we recommend creating a PDF file that is zoomable without loss of quality because it is vector-based. It follows the code of Fig. 1, an example of a customized nested chart on a hypothetical superordinate construct of Openness (O) and Extraversion (X). More details and code examples can be found in the vignette (browseVignettes(package = "IPV")) and the documentation of the package (?IPV).

This code generates the default version of the chart:

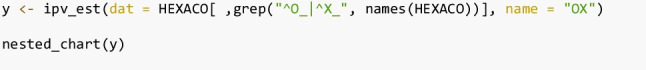



Given an extreme variation in the center distances, some default versions of charts may be practically unreadable without zooming. Therefore, the smallest elements and fonts should be increased in size while overlap should be avoided. It is also possible to show only the relevant section of the chart. This is the code that generates the version shown in Fig. 1:

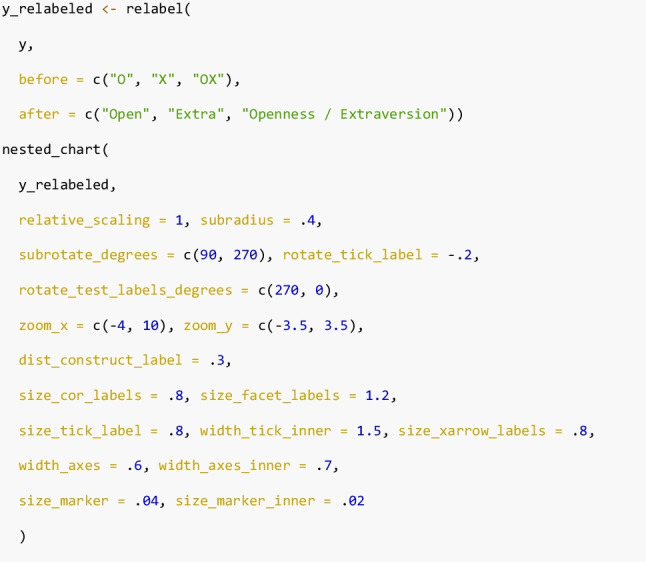



The relabel function allows the user to change any factor or item label in the estimates. The area shown is restricted by zoom_x and zoom_y to increase the display size. The argument relative_scaling determines how much the facet charts, which are plugged into the circles of the overall chart, get rescaled. This enables users to cope with cases where center distances are much larger (or smaller) on the global level, compared to the nested level. The desirable relative scaling of 1 indicates that the axis scale is equal for both the global chart and the nested charts within its circles. Although the dotted circles indicating the axis scaling adapt automatically, it is prudent to inform readers whenever the relative scaling is not 1 to avoid misinterpretation. Several width_... and size_... arguments make it possible to keep individual elements of the chart small enough to not overlap with each other and large enough to be readable. The global argument size scales all of these at once. Using rotation arguments is especially useful to straighten out correlation arrows and avoid overlap. Here, the (arbitrary) size of the innermost circles (subradius) was set large enough to make labels and values within them readable. If the display of color is possible, it is recommended to increase visual clarity.

Calling file_name = some_file_name.pdf in the ..._chart function saves a vector-based, high-quality, zoomable PDF version of the chart. The pixel-based formats .png and .jpeg are supported as well. The parameter dpi = can be used to change the resolution of pixel-based files. To retain the vector-based quality of PDF files in partial displays it is recommended to use zoom_x and zoom_y.

The IPIP HEXACO equivalent scales define X more narrowly than O. This is indicated by the relatively compact inner structure of the Extraversion (X) circle compared to the Openness (O) circle (see Fig. 1). Within the O circle Creativity (Crea) is most central, which indicates that O is almost equivalent to this facet in its power to explain the facet’s item variance. The facet Aesthetic Appreciation (AesA) is displayed much further away from the center. Representing its items by AesA uncovers that a substantial amount of content is specific to the items of this facet. The dotted arrows show that two facets of X (Liveliness and Social Boldness) have a distinctly strong relationship with the Creativity facet of O. The correlations between these facets somewhat exceed the correlation between X and O. This example shows that IPV allows more precise interpretations of factor correlations. The result “there is a correlation between X and O” can now be further specified as a particularly strong relationship of Creativity (O) with Liveliness and Social Boldness (X). The other facet correlations across personality traits O and X are weaker. Some are close to 0:

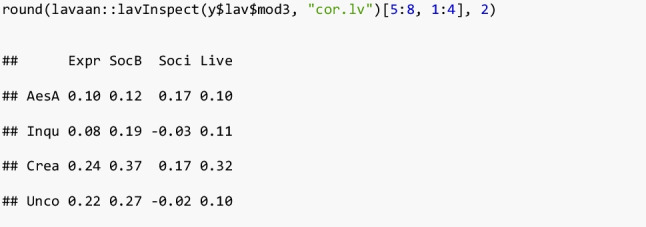

- **Overview of factor loadings.** IPV charts provide an overview of the relative increase in squared factor loadings using a specific compared to a broad model. The item_overview function of the IPV package provides an overview of all the involved absolute (squared) factor loadings (y-axis). This helps to identify general patterns in the factor loadings and makes the calculation of center distances transparent. By default, the factor loadings are squared to reflect the explained variance from which center distances are calculated in IPV.

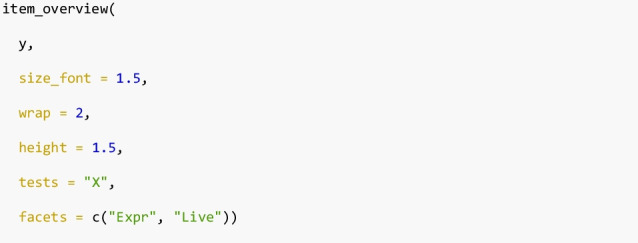

**Fig. 6:**
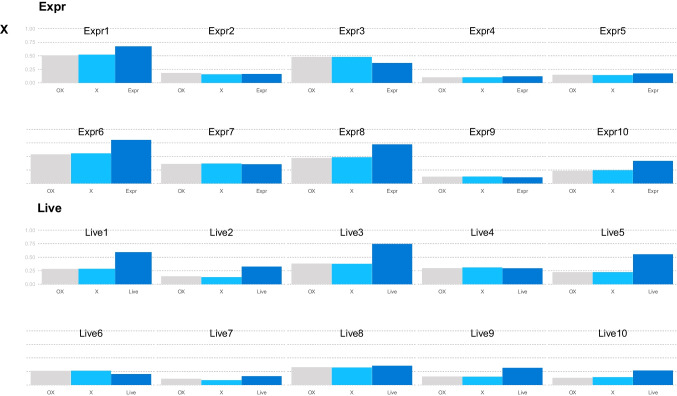
Squared factor loadings (= explained item variance) of all three models of the HEXACO openness and extraversion example. Only a subset of the facets is shown for readability; a complete figure can be found in the Appendix (Fig. 7). X = Extraversion, Live = Liveliness, Expr = Expressiveness

Figure 6 shows some of the squared factor loadings from the Extraversion/Openness model above^4^. The items are ordered by personality trait and facet. For each item there is a small bar chart with three bars representing the squared factor loadings in the three different models. On the x-axis, the name of the respective factor is indicated. The bar colors indicate the model—analogous to the colors in the IPV nested chart (Fig. 1). There is substantial variation in the absolute factor loadings that is not visible in relative comparisons. For example, the Expressiveness facet of Extraversion (Expr) contains several items with high and several items with low factor loadings in all three models. In the nested chart the Extraversion items showed similar factor loadings on the combined “OX” factor as on the factor “X”, indicated by a low center distance in Fig. 1. The overview provided in Fig. 6 thus shows that the loadings on the “OX” factor and the “X” factor are almost identical on the Expressiveness and Liveliness facets. The consequence is a near-zero aggregate center distance of “Extra” (version) (Fig. 1).

Although highly desirable for the advancement of psychological measurement, comprehensive data on several similar measures applied to the same sample (as in Dantlgraber et al., 2019; Frey et al., 2017; Moshagen et al., 2020) are rarely available—a gap that future research should fill.

## Conclusion

The IPV package makes the use of IPV charts accessible. Its aim is to allow researchers to add IPV to their repertoire with minimal effort. Creating IPV charts from raw data requires two simple function calls, because the package streamlines model specification, model estimation, and chart creation. In addition, it improves IPV conceptually by introducing the aggregate center distance and the item overview chart. It provides many customization options and generates high-quality, vector-based PDF output.

Given the necessary data, IPV guides choices between the many available measures. It informs the understanding of measures in the presence of inconclusive terminology (“jingle-jangle”). In the example, IPV uncovered that the facets of HEXACO traits show varying degrees of diversity—both between facets and between items within facets. Specifically, IPV shows which facets and items show the most variance that is unique to the facet and not captured by the overall trait. In addition, trait correlations can be explained by specific facet correlations. For example, the correlation between Openness and Extraversion could be further specified as a particularly strong relationship of Creativity (O) with Liveliness and Social Boldness (X), while the other facet correlations are weaker. In this way, the R package IPV facilitates a more differentiated understanding of human behavior.

## Figures and Tables

**Fig. 7:**
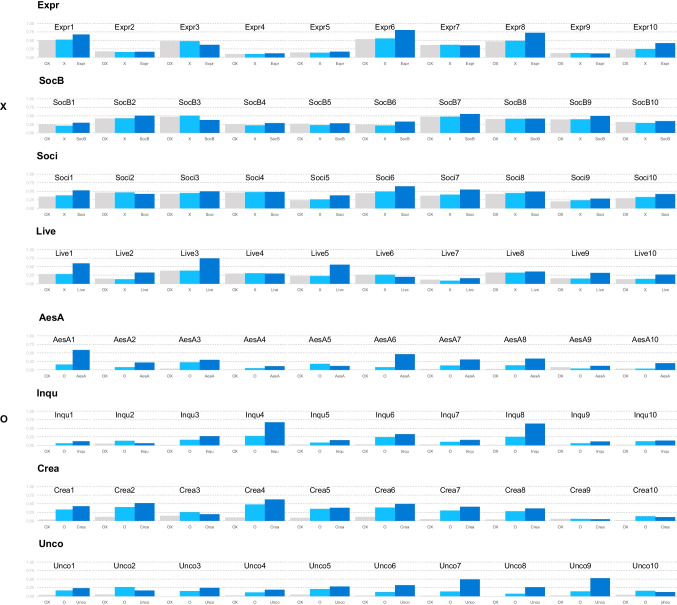
Squared factor loadings (= explained item variance) of all three models of the HEXACO openness and extraversion example data; O = Openness, X = Extraversion, AesA = Aesthetic Appreciation, Inqu = Inquisitiveness, Crea = Creativity, Unco = Unconventionality, Soci = Sociability, Live = Liveliness, Expr = Expressiveness, SocB = Social Boldness

## Appendix

Complete item overview

## Footnotes

1 Note that item variances are standardized in the calculation of the factor loadings, so variations in item variance have no effect on aggregate center distances.

2 As described in the documentation of lavaan, see ?lavaan::lavOptions.

3 Alternatively, the raw data can be provided in long format, as described in the package documentation.

4 A complete overview can be found in the Appendix (Fig. 7).
